# COVID- 19 and human right to food: lived experiences of the urban poor in Kenya with the impacts of government’s response measures, a participatory qualitative study

**DOI:** 10.1186/s12889-022-13638-3

**Published:** 2022-07-21

**Authors:** Elizabeth Wambui Kimani-Murage, David Osogo, Carolyn Kemunto Nyamasege, Emmy Kageha Igonya, David Otieno Ngira, John Harrington

**Affiliations:** 1 Department of Nutrition and Food Systems; African Population and Health Research Center, APHRC Campus, 2nd Floor, Manga Close, Off Kirawa Road, P.O. Box 10787-00100, Nairobi, Kenya; 2 International Health Institute, Brown University, Providence, RI USA; 3 Department of Clinical Trials and Clinical Epidemiology, University of Tsukuba, 1-1-1 Tennodai, Tsukuba, Tsukuba, Ibaraki 305-8575 Japan; 4 Cardiff University, Law Building, Museum Avenue, Cardiff, Wales CF10 UK

**Keywords:** COVID-19, Response measures, Right to food, Vulnerable populations, Kenya

## Abstract

**Background:**

Globally, governments put in place measures to curb the spread of COVID-19. Information on the effects of these measures on the urban poor is limited. This study aimed to explore the lived experiences of the urban poor in Kenya in the context of government’s COVID-19 response measures and its impact on the human right to food.

**Methods:**

A qualitative study was conducted in two informal settlements in Nairobi between January and March 2021. Analysis draws on eight focus group discussions, eight in-depth interviews, 12 key informant interviews, two photovoice sessions and three digital storytelling sessions. Phenomenology was applied to understand an individual’s lived experiences with the human right to food during COVID − 19. Thematic analysis was performed using NVIVO software.

**Results:**

The human right to food was affected in various ways. Many people lost their livelihoods, affecting affordability of food, due to response measures such as social distancing, curfew, and lockdown. The food supply chain was disrupted causing limited availability and access to affordable, safe, adequate, and nutritious food. Consequently, hunger and an increased consumption of low-quality food was reported. Social protection measures were instituted. However, these were inadequate and marred by irregularities. Some households resorted to scavenging food from dumpsites, skipping meals, sex-work, urban-rural migration and depending on food donations to survive. On the positive side, some households resorted to progressive measures such as urban farming and food sharing in the community. Generally, the response measures could have been more sensitive to the human rights of the urban poor.

**Conclusions:**

The government’s COVID-19 restrictive measures exacerbated the already existing vulnerability of the urban poor to food insecurity and violated their human right to food. Future response measures should be executed in ways that respect the human right to food and protect marginalized people from resultant vulnerabilities.

## Introduction

### Background

Coronavirus disease 2019 (COVID-19) was declared a pandemic by the World Health Organization (WHO) on March, 112,020 [1]. Governments enforced control strategies to “flatten the infection curve” [2], in response to measures suggested by the WHO and US Centers for Disease Control (CDC) [3]. For instance, governments restricted international travel through border shutdowns, strict quarantine measures, curfews, closures of businesses, restricting social and sporting activities likely to generate crowds among many other measures [4].

A majority of low and middle income countries (LMICs), which have adverse social determinants of health and system challenges, quickly responded to the pandemic by implementing the range of COVID-19 control measures. For instance, India was initially praised by the WHO for being ‘tough and timely’ in declaring a nationwide lockdown that affected 1.3 billion persons [5]. However, these measures were later criticized, given that India was subsequently reported as being among the top three most infected countries and one of the most affected in regards to food insecurity and hunger [4]. Sub-Saharan African countries such as Ghana, Sudan and South Africa also responded quickly to contain the spread of COVID-19 by implementing lockdown measures [6]. Equally, the government of Kenya promptly implemented measures such as the suspension of international flights, partial lockdown, school closure, curfew, compulsory wearing of masks, a ban on social gatherings, restriction of business operating hours, social distancing and cessation of movement within cities following a marked rise in the COVID-19 cases [7, 8]. Incidentally, these restrictions aimed at mitigating the pandemic have been shown to contribute to vulnerabilities such as strained socioeconomic activities, uncertainty, anxiety, mental distress, loss of livelihoods and violation of human rights as reported in previous studies [9, 10]. Moreover, the attendant disruption of food systems has heightened food insecurity while the financial power to access food has been affected as reported in a study conducted in Uganda and Kenya [11].

A report by the United Nations (UN) on Covid-19 states that governments may impose limits on certain human rights in order to respond to national emergencies. Indeed this is provided for by international human rights law [8, 12]. The UN has affirmed that response measures shaped by respect for human rights are likely to lead to better outcomes in overcoming pandemics, preserving human dignity and ensuring healthcare for all [13].

Food is a human right, recognized under Article 25 of the Universal Declaration of Human Rights (UDHR) as part of an adequate standard of living [14]. This was expanded upon in Article 11 of the International Covenant on Economic, Social and Cultural Rights (ICESCR), which establishes the inherent human right to adequate food, [15]. Further, the Committee on Economic, Social and Cultural Rights (CESCR) in General Comment No. 12 (1999) has defined the right to adequate food as “the right to feed oneself and one’s family with dignity, through sufficient availability, accessibility, and adequate fulfilment of dietary needs in a sustainable manner”. This is realized “when every man, woman and child, alone or in community with others, has physical and economic access at all times to adequate food or means for its procurement” [16].

The right to food is enforceable in Kenya, on the basis of the ICESCR, which it has ratified and incorporated into domestic law, and also Article 43 (1c) of the Constitution of Kenya, 2010 (COK, 2010) which stipulates that: “every person has the right to be free from hunger and to have adequate food of acceptable quality”. Other related articles in the COK supporting right to food include Article 53 (1c). As provided for by Article 21 of the COK, which reproduces the CESCR approach in General Comment No.12, the State’s duties in relation to the right to food are to observe, respect (not interfere with one’s ability to acquire food), protect (ensure others do not interfere with one’s ability to acquire food) and fulfil (either provide an enabling environment for food production or procurement or directly provide food to those who are not able to produce or procure food for themselves and their families due to loss of livelihood, conflict, detention, natural disasters or other reasons) [17, 18].

Notwithstanding the above, the right to food continues to face normative challenges. For instance, the domestication of article 2 of the ICESCR under article 21 (2) of COK which imposes a duty of ‘progressive realization’ rather than immediate fulfilment in relation to socio-economic rights’ presents a significant loophole by which the Kenyan authorities may evade their obligation to secure the right to food to citizens. The doctrine of ‘progressive realization’ is based on the notion that the state does not have limitless resources and that, as a result, it needs only to show that it is working towards the full realization of the right in question, [19]. This doctrine makes it less likely that the right to food will be justiciable notwithstanding the existence of constitutional protections under article 43 (1) (c). Moreover, the principle of progressive realization also presumes that countries with greater resources have a more substantial obligation to realize socio-economic rights than poor ones, and that the scope of a country’s obligations expands with its increase in economic development (Ibid: 6) [19]. That has not been true of Kenya, where significant economic development over the last 20 years has not resulted in any corresponding increase in the protection of the right to food.

Despite the right to food being recognized as a basic human right by most countries (with Australia and the United States of America as notable exceptions), during the Rome Declaration on World Food Security in 1996 [20], many people globally are food insecure. In 2020, about 720–811 million people worldwide, mostly women and children were hungry while 2.37 billion were food-insecure (defined as a lack of consistent access to enough food for every person in a household to live an active, healthy life) [21, 22]. This problem is also evident in Kenya. Previous research indicated that majority (over 80%) of the households in the urban informal settlements of Nairobi were food insecure [23]. A report on the threat posed by the pandemic to food security states that the three elements of the rights to food (availability, accessibility, adequacy) as recognized by the UN Committee on Economic, Social and Cultural Rights, and the four pillars of food security (availability, accessibility, stability, adequacy/utilization) have been affected by measures taken to stop the spread of COVID-19 [24]. Consequently, this threatens the achievement of the UN Sustainable Development Goals (SDGs) such as ending poverty, achieving zero hunger, good health and wellbeing, among others [25]. Moreover, as the UN Committee has indicated, ensuring freedom from hunger is a core obligation of states, not subject to the doctrine of progressive realization. As a result, it must be discharged by the state as a matter of priority making every effort possible [16].

As with all crises, the consequences of the COVID-19 outbreak are felt most acutely by those already marginalized in society. Information on their experiences is essential to understanding the extent to which basic human rights, guaranteed nationally and internationally, have been secured. Moreover, the value of participation is a cornerstone of Kenya’s constitutional system, and recognized as an essential element of international human rights [26, 27]. Yet studies reporting on the vulnerability of the urban poor and their lived experienced regarding their right to food in the context of COVID-19 restrictive measures are limited. In filling this gap, the present study was conducted in urban poor settings in Kenya during COVID-19. Our research was guided by the human rights framework regarding the right to food, set out above. We focussed on the three human right to food elements [16], namely, availability: whereby food should be available from production by cultivating land or animal husbandry, from natural resources, fishing, hunting and gathering; accessibility by ensuring economic accessibility in terms of affordability without compromising other basic needs and physically accessible to all, including the vulnerable in the society; adequacy to meet an individual’s dietary needs while considering a person’s age, sex, living conditions, occupation, health, etc. The study aimed to explore the experiences, perceptions, and attitudes of vulnerable citizens in the informal settlements of Nairobi with regard to the impact of the government’s COVID-19 response measures on enjoyment of the human right to food.

### Kenyan government’s COVID-19 response measures and intervention programs

Table 1 below shows the government’s COVID-19 response measures and the dates when they were implemented. The government of Kenya implemented a partial lockdown on 6 April 2020 with only 158 COVID-19 reported cases and 6 deaths. Moreover, schools were temporarily closed on 13 March 2020, 2 days after the first reported case, a mandatory quarantine of incoming residents in designated centers was imposed, international flights were suspended, large gatherings and restaurant-opening hours were restricted on 25 March 2020, bars were temporarily closed, followed by a nationwide curfew from 7p.m to 4 a.m. and restricted movement from and to Nairobi Metropolitan Area. Further, the Kenyan government declared compulsory wearing of face masks in public and cessation of movement out of Nairobi and Mombasa, and later in additional five counties across Kenya, following a marked rise in the COVID-19 cases [28].

**Table 1:** Response measures

Measure	Start date	End date
Daily curfew	27th March 2020	20th October 2021
Wearing of face masks	6th April 2020	11th March 2022
Ban on public gatherings	27th March 2020	11th March 2022
Social distancing	27th March 2020	11th March 2022
Movement restrictions (lockdowns)	16th March 2020	7th July 2020
Economic relief measures (tax relief and reduction of income tax, reduction of turnover tax, appropriation of cash to elderly and other vulnerable persons through cash transfers, temporary suspension of Credit Reference Bureau (CRB) listing, reduction of VAT from 16 to 14%, payment of pending bills, payment of verified VAT claims, lowering of Central Bank lending rates from 8.25 to 7.25%)	25th March 2020	1st January 2021 (for tax measures) 27th November 2020 (for cash transfers)
Encouragement of work from-home framework	27th March 2020	

Other restrictive measures included social distancing, where people were encouraged to stay at home and to avoid shaking hands or hugging, or meeting in large numbers, for example in social gatherings such as weddings and burials [7, 8]. In addition, the government implemented other measures such as provision of water tanks, taps and soap for hand washing and hand sanitizers in the community particularly outside business premises, and strict observance of proper sanitation and hygienic practices.

Alongside the primary COVID-19 response measures, the government in partnership with Non-Governmental Organizations (NGOs), faith-based organizations, private stakeholders, and well-wishers put in place measures/programs to promote compliance with preventive measures and to cushion/mitigate the unintended consequences of the restrictive measures. The programs were: economic relief (Table 1) provision of cash transfers, food aid, revival of neighbourhood watch committees, strengthening social safety nets, establishment of economic empowerment initiatives such as a youth program called Kazi Mtaani, that provided cleaning jobs for jobless youths, provision of personal protective equipment (PPE), and COVID-19 sensitization efforts [11].

To ensure compliance, strict enforcement measures using police, village elders, chiefs, and heads of “Nyumba Kumi Initiative” (a government strategy to complement community policing at household level) were used. In enforcing response measures police were widely reported to have used excessive force particularly in informal settlements. Other forms of police harassment including intimidation and bribes also characterized this period [29].

## Methodology

### Study design and approach

This was a qualitative exploratory study aimed at documenting the lived experiences of community members. Phenomenology was applied to understand an individual’s lived experiences concerning the human right to food during COVID − 19. Through the phenomenology approach, authors seek to describe the essence of a phenomenon from the perspective of those who have experienced it, with a goal of describing the meaning of their experience in terms of what and how it was experienced [30]. Specifically, Dahlberg’s reflective life world approach was applied [31]. Both hermeneutic (interpretive) and transcendental (descriptive) phenomenology were applied during data collection, analysis, and presentation of the results.

### Study settings

This study was carried out in Korogocho and Viwandani, low-resource informal settlements, in Nairobi, Kenya [32] from January –March 2021. The two urban informal settlements are about seven kilometers apart covering a total area of approximately one km^2^ with about 89,000 individuals from 33,500 households [33]. The informal settlements are densely populated with more than 60,000 inhabitants per square km and are characterized by poor housing, lack of basic infrastructure, violence, insecurity, high unemployment and poverty rates, food insecurity, and poor health and nutrition indicators [32].

### Study population and sampling

The study participants were residents of Korogocho and Viwandani and selected community leaders including area chiefs, village elders, ward representatives, and senior health officials. These participants were conveniently selected so as to ensure a fair representation from the communities. Selection of participants for Focus Group Discussions (FGD), photovoice and digital stories was based on their knowledge and experience in the government’s response measures to COVID-19 in urban informal settlements. In addition the selection of participants of In-depth Interviews (IDI) and Key Informant Interviews (KII) was based on the perceived critical role played by interviewees in these areas. Convenience and purposive sampling technique was used in identifying eligible participants. A consecutive sample of older and younger male and female community members was selected in order to ensure representativeness with regard to age and gender.

### Data collection methods and sample size

Qualitative techniques: FGD, IDI, KII, and participatory research methods, mainly photovoice and digital storytelling were employed in data collection to understand, and document lived experiences of the participants. A total of eight FGDs, each involving a total of six homogeneous participants were conducted: four in each site (Korogocho and Viwandani). The participants included separate groups of women, men, youth, and traders in each study site. In addition, a total of eight IDIs were conducted, four in each site. The IDI participants were adult men and women. In addition, 12 KIIs were conducted with community leaders (chiefs, village heads, ward administrators, religious leaders, Community Health Volunteers (CHVs), and public health nurses (PHN), six in each site.

Participatory methods such as photovoice and digital stories were also used to document the lived experiences of community members as regards the government’s COVID-19 response measures. Through photovoice, a participatory and visual research methodology, participants used photography to identify, capture, and express issues with respect to certain aspects of their lives [34]. Participants produced a short video clip through digital storytelling, another participatory method for telling their story in a compelling and emotionally engaging and interactive format [35]. A mixed group of both youth and adults was engaged in each site for the photovoice activity, with each session having six participants. A similar group was engaged in each site for digital storytelling activity.

Photovoice participants received cameras and were trained in basic photography skills by the research team. They were asked to take photographs that depicted their perspectives and lived experiences as regards government COVID-19 measures and the impact on their lives. Thereafter, they developed captions for their photos and held discussions to tell the stories depicted in their photos. In digital storytelling, participants were guided through scripting and development of their stories, narration, and shooting (recording), and editing to produce a good story. The stories were then screened in a session for the participants themselves to allow for criticism and improvement, ahead of the production of final versions.

### Data analysis

Data was analyzed thematically based on the right to food framework [27]. Familiarization with the data began in the field, evolving together with data collection, and it involved various steps based on thematic analysis of qualitative data [36]. Firstly, a review of the collected data was done (transcripts and audio) by the qualitative field interviewers through field debrief sessions at the close of each interview day. These sessions allowed the team to jointly identify and document emerging salient topics and themes in relation to the research objective. A debrief form reflecting the research question was developed for reference. Secondly, all interviews and photovoice discussions were transcribed verbatim in word format.

Individual transcripts were thoroughly read and scrutinized by the data analysis team while identifying and creating codes based on relevant meaningful patterns across respondent groups. The identified responses relevant to the research question were clustered according to similarities while subthemes were categorized inductively. Three themes were identified namely: lived experiences of the impact of government’s COVID-19 response measures on livelihoods and food security; the human right to food as regards food availability, accessibility, and adequacy; social protection and coping strategies in regard to food acquisition. Further, an exhaustive, condensed description of the responses was generated. Coding was conducted by two researchers and a code book was generated and shared with all the research team for reference and agreement with the coding. Data organization and coding was performed using NVIVO, a Qualitative Data Analysis (QDA), software [QSR International Pty. Ltd., Melbourne, VIC, Australia] (release 1.5 for Windows).

### Ethics approval and consent to participate

Ethics approval for this study was provided by the Amref Health Africa Ethics and Scientific Review Committee (ESRC). Permission for community entry was sought from the local authorities and community level gatekeepers. The ethical principles guiding research in human subjects in accordance with the Helsinki Declaration, including respect for human autonomy, beneficence, non-maleficence, and justice, were followed. Participants for both qualitative and participatory method-based studies voluntarily accepted to take part in the study. Written informed consent was sought from each of the participants before conducting the interviews and a verbal informed consent for phone interviews. Photovoice participants provided consent before photos and videos were taken. No images of faces were taken, nor was identifying information as regards to people, specific premises or structures retained. Unique identifiers and pseudonyms were used to ensure confidentiality. Participants were given a small allowance to compensate them for transport costs and their time.

Adherence to government protocols and the research institution’s data collection guidelines in the context of COVID-19 pandemic were observed during data collection. Further face-to-face engagements were conducted in open spaces and in spacious and well-ventilated rented rooms so as ensure adherence to social distancing guidelines (at least 1.5 m between the respondent and the interviewer). Researchers provided hand sanitizers and face masks to all participants and data collectors during face-to-face interviews.

## Results

### Characteristics of the study participants

A total of 80 adults participated in this qualitative study. Forty-eight people participated in the FGDs, eight in the IDIs, 12 in the KIIs and 12 in the photovoice. However, personal characteristics of 17 participants’ (12 KIIs and 5 IDIs) were not collected. Out of the 63 participants, their age ranged from 18 to 58 years with a mean (SD) age of 37.1 (12.7). Most of the respondents were either married (50.8%) or single (38.1%), had primary (46.0%) or secondary school (42.9%) level of education and were either doing business (47.6%) or casual labor (27.0%) while most of the youth (< 24 years old) were either casual labors (29.4%) or unemployed (38.5%). The proportion of male (49.2%) and female (50.8%) participants was almost the same, Table 2.

**Table 2:** General characteristics of the participants

Participants’ characteristics	Total ***N*** = 80	
	n	%
**Participants missing individual data**	**17**	**21**
**Participants with individual data**	**63**	**79**
**Village of residence**		
Korogocho	30	47.6
Viwandani	33	52.4
**Age group**		
< 24 years old	14	22.2
25–35 years old	16	25.4
36–55 years old	26	41.3
56 years and above	7	11.1
**Gender**		
Female	32	50.8
Male	31	49.2
**Education level**		
Elementary school	29	46.0
Secondary school	27	42.9
College/university	7	11.1
**Occupation**		
Unemployed	13	20.6
Casual labor	17	27.0
Own business	30	47.6
Employed	3	4.8
**Marital status**		
Married	32	50.8
Single	24	38.1
Divorced/separated	4	6.3
Widowed	3	4.8
**Religion**		
Christian	58	92.1
Muslim	5	7.9
**Ethnicity**		
Kikuyu	20	31.7
Luo	13	20.6
Luhya	12	19.0
Kamba	8	12.7
Kisii	6	9.5
Borana	4	6.3

### Lived experiences with regards to food security and the human right to food

The government’s COVID19 response measures were reported to have serious impacts on the livelihoods of the urban poor residents due to loss of jobs, and disruption on business. As the following shows, the impact of the measures on livelihoods consequently had a dire impact on food security and the right to food. Additionally, food security and the right to food were directly affected by the government response measures. Firstly, the authors describe the impact of response measures on socioeconomic status, and a spillover effect on food security, focusing on food availability, food accessibility and food adequacy, highlighting how the right to food was violated. Secondly, results are presented on mitigation measures put in place by the government and partners to alleviate the impacts on food security and the right to food and the coping strategies employed by the community regarding food acquisition.

### Livelihoods

**Fig. 1:**
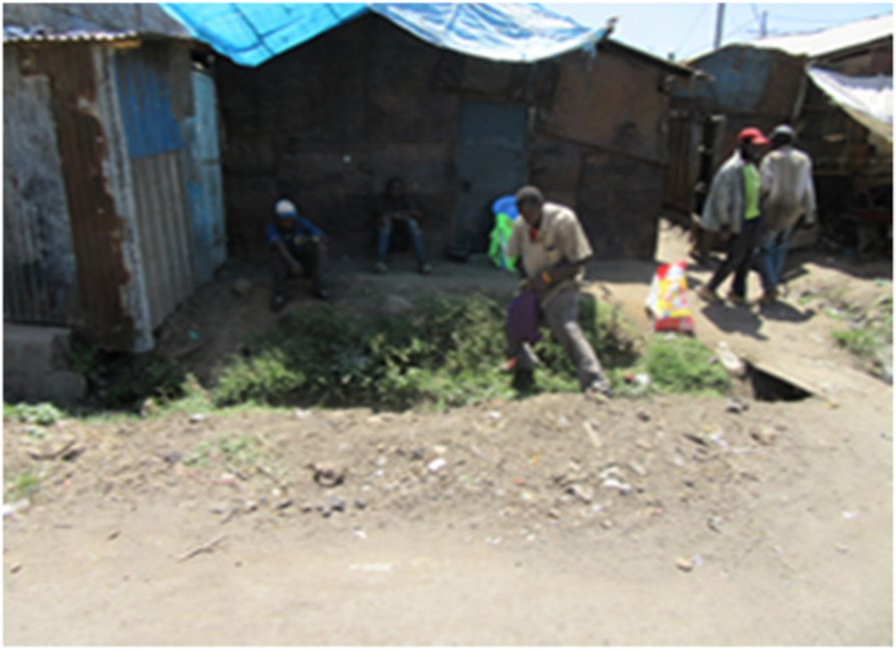
Photo 1 showing situation regarding livelihoods in Nairobi slums during COVID19 lockdown period; Photo Credit: APHRC

Participants suggested jobs/employment (including causal labor/wages in local factories), businesses (petty or small business) and scavenging food from the dumpsite were the main facilitators of food acquisition in the two study sites. A lot of people living in the informal settlements work in the industries in these settings. The study suggests that these industries were forced to reduce staff to comply with social distancing and curfew measures and to respond to reduced demand due to interruption to the market and supply chain. Many other employers also sent their employees home due to the measures and related disruptions. In addition, lockdown and social distancing limited casual laborers from accessing their area of work. Employers feared getting infected in the case of dayshift house servants. Consequently, many people lost their livelihoods because of loss of jobs and disruptions caused by these measures Fig. 1.

“... This is the effect of COVID-19 and as you can see in the picture people are idle. So, people have lost their jobs so the picture represents how people are idle in the community.” (Photovoice, mixed participants, Korogocho).

Thirty-two-year-old Purity K (pseudonym), a mother of four children and a primary school dropout explains her experience of jobloss when COVID-19 struck in her digital storytelling [37]:

“My parents were not able to pay my school fees, so I dropped out of school in class seven. When I was 23 years, I was lucky to get a job at a factory. Before the corona pandemic was in the country, life was not so hard. We had enough food to eat, clothes to wear, there was happiness in my family. After the pandemic [struck], life became harder. The factory laid me off due to COVID 19 measures like social distancing. Life became hard because of the responsibilities, and I did not have money to care for my children. I did not have money to pay rent. Eventually, my neighbors took my children to feed them because I was not in a position to do so. Now that life is a little bit back to normal, I tried going back to my old job however due to lack of education; I did not manage to get my old job back. People who have higher education than me and had previously lost their jobs are the ones who are doing my job right now”. (Purity, digital storytelling speaker 1).

Purity’s experience resonates with that of other research participants suggesting the impact of COVID 19 through loss of employment which affected access to food. In her digital storytelling, Mama Wangechi (pseudonym), married with four children living in Korogocho tells her predicament with coronavirus [38]:

“Before corona, my husband used to work at an industry in Baba Dogo. Life was good before corona. Ever since the pandemic, life changed. My husband lost his job at the industry because the company reduced its production and the government put in measures for example social distancing [ …] Food became hard to find due to change in income […] The situation at home worsened and […] the arguments at home every day led to my husband leaving and I was left alone with the children” (Mama Wangechi, digital storytelling speaker 2).

Moreover, many businesses were also shut down in compliance with the regulations while some had to close due to persistent losses. Curfew hours affected the many businesses who are normally busiest in the evening and at night, when customers coming from work look to buy provisions. In addition, cessation of movement and border restrictions disrupted supply chains, which also affected businesses. The reduced purchasing power of the customers which ensued, also negatively affected businesses.

“...Businesses were affected because there were shops that were closing at eleven or ten but when the curfew was imposed, they couldn’t close late and that affected their business which has reduced their incomes...” (Photovoice, mixed participants, Viwandani).

“...Personally COVID-19 has really affected us because my husband lost his job, the business I had was also affected because the customers also lost their jobs, so they didn’t have money – the few that are there all ask for debts and they don’t even repay it sooner. You need food but don’t have money to buy the food so it really affected us.” (FGD, adult females, Viwandani).

### Food security and violation of the right to food

Participants narrated the way in which government response measures impacted on their food security and violated their human right to food. Loss of livelihoods and measures restricting movement compromised access and food supply respectively, and resulted in limited access to safe, adequate, nutritious food, and therefore violated the right to food.

“…A child has the right to food and humans have right to food, but you don’t have the means to look for the food so that right is already violated. So, unless they would get these organizations to support, and they wouldn’t support the whole community so some people would still miss out. That was discrimination on the right to food” (FGD adults female Korogocho).

In her digital storytelling, Mama Wangechi’, noted how their family moved from three meals a day to water only [38].

“Earlier [before COVID 19] we used to have meals thrice a day, but things changed since Corona came. We used to drink tea with milk but nowadays we drink hot water …. or borrow from the shops and from the vendors in the neighborhood but not every day because they would not accept” (Mama Wangechi, digital storytelling speaker 2).

Participants in the FGD consisting of traders reported that food vendors had their small businesses closed by the local authorities for not complying with hygienic measures such as provision of water and soap for customers to wash hands before being served. Moreover, police prevented people from accessing food within the curfew times or those not observing the restrictive measures. Besides, with loss of livelihoods, food prices were unaffordable to community members. The FGD participants intimated that the government, in not intervening to reduce food prices, failed to respect and protect the vulnerable on their right to food.

While in the perspective of ordinary community members the measures interfered with human rights, local leaders had a different view. For them, existence, above all is at the core of human rights. Thus, they considered that the government had upheld human rights by virtue of the fact that it moved quickly and irresolutely to put in measures against the very real threat posed by COVID-19: ie. the loss of human lives from infectious disease.

The rest of this study further suggests how the different aspects of the right to food: food availability, food access and food adequacy were affected by the government COVID19 response measures.

### Food availability

Curfew and lockdown, particularly restrictions on movement, were government measures highlighted by many participants as negatively affecting food availability. The quantity and variety of food sold by community vendors in the market were both affected. For instance, curfew limited traders from accessing the wholesale markets early enough, normally at dawn, to obtain the best produce in terms of quality, variety, and lower prices. The food supply chain was also disrupted by the national lockdown, border shutdown, cross-border restrictions such as requirement of COVID 19 tests and certificates, long queues, high cost of transportation, and the cumbersome requirement to obtain expensive permits for transporting food from rural to urban settings due to the restrictions on movement. As a result, FGDs, KII and photovoice participants suggested that the supply of food, mostly vegetables, fruits and cereals including those imported from neighboring countries was limited, considering their perishable nature.

You know there were people who transported the foods from upcountry to sell here and when the curfew was imposed it was expensive to apply for travelling letters and the cost of food had to increase. So only a little food was available, and it was also expensive” (FGD, traders, Viwandani).

**Fig. 2:**
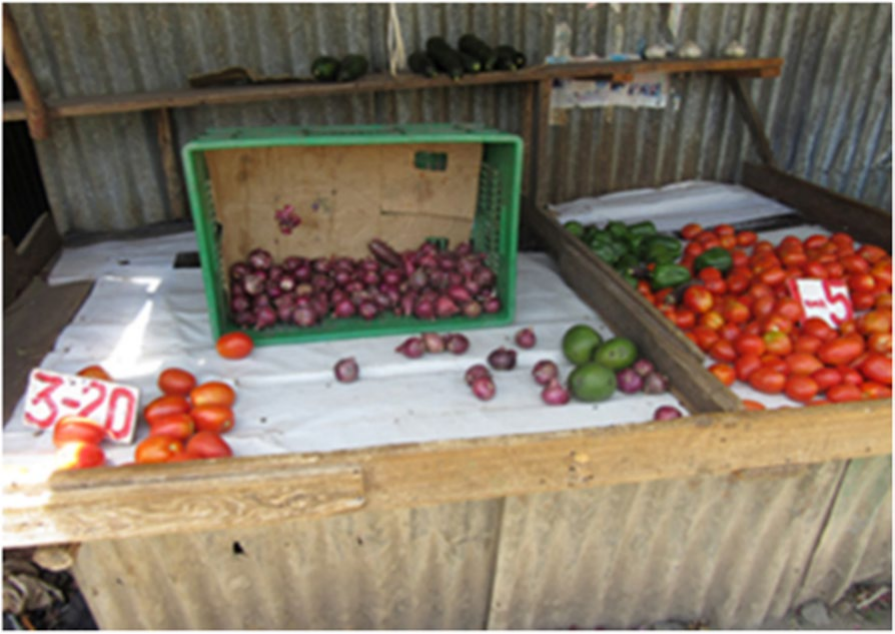
Photo 2 showing the situation of food availability in the markets in Nairobi slums during COVID19 lockdown period; Photo Credit: APHRC

Moreover, given the lower volume of sales, some vendors reduced the quantity of food to avoid making losses, while others closed their businesses owing to fear of contracting COVID-19. Furthermore, many people left Nairobi for rural areas, which further reduced the turnover of local stores Fig. 2**.**

“...Businesses reduced because people were few in the community and those who were working also reduced so business was really affected. If you look at the photo you will see the market is almost empty. So, businesses went very low...” (Photovoice, mixed group, Viwandani).

Likewise, suspension of international flights and closing of hotels interrupted the availability of free and cheap food from the nearby Dandora dumpsite, the largest in the city.. (Residents and traders normally scavenge leftover food such as yoghurt, fish, milk, bread, sugar, burgers, disposed of by airlinesen route to Nairobi.

“Sometimes we do get fish or yoghurt that is dumped when the planes fly in, and they are left in the planes.” (FGD, adult males, Korogocho).

In addition, some food commodities, mostly those sold in supermarkets, went out of stock as a result of panic-buying by better-off customers. It is worth noting, however, that some participants acknowledged that most of the food commodities were generally available though expensive.

### Food accessibility

Participants narrated that majority of the urban informal residents access food through purchase. They also live on hand to mouth basis with minimal or no savings at all and mostly depend on small businesses and casual labor as a source of livelihood. The response measures such as lockdown and curfew led to limited livelihoods. This constrained food accessibility due to limited purchasing power. Food prices also rose due to limited availability in the market.

“...The prices of things that people use daily such as food, water and soap were hiked due to the curfew and change in business hours due to COVID-19. So, you find that if you were buying a sack of maize at two thousand shillings, now it costs two thousand five hundred because they (vendors) have risked bringing it from the farms and the measures also make it such that if they were to bring five sacks of maize in a day, they end up bringing two or three because of cessation of movement during curfew time. So, the products found in the shops also had their prices hiked because the transportation time has been reduced….” (Photovoice, mixed group, Viwandani).

During normal times, some community members normally buy their food at distant wholesale markets where the prices are lower, some of which require the use of minibus taxis (Matatus) to get to the markets. The Matatus increased their fares to make up for reduced passenger load as they were restricted to carrying only 60% of the normal number.The cost of accessing preferred markets therefore went up, acting as a disincentive to regular customers, with many resorting to walking or opting out of this traditionally popular option. As such, the accessibility of food through this channel was also disrupted.

“...It was a challenge because you would find that kales were cheaper in Muthurwa (wholesale market) but transport cost was double so you would have to walk to buy the cheaper kales. So, the high transport cost also made it a challenge….” (FGD, adult males, Korogocho).

The curfew restricted business hours, severely limiting people’s access to food while kiosks and markets were shut. Some people would get back home late in the evening after a long day of work with the intention of buying food for their families only to realize that all businesses had been closed. Such people would go hungry, not because of lack of money but because food was not there at the time they returned from work. Curfew times therefore hindered access to food for some people owing to the nature of their jobs.

“...Also, you would find that for the vegetable sellers, I would (for example) have money after doing my job and earning from it but I cannot buy food because of the curfew, and they (food vendors) have closed. So, I would sleep hungry and yet I have the money...” (FGD, adult males, Korogocho).

Moreover, family separation because of domestic violence aggravated by unemployment, the inability of the household head to provide for the family and idleness, increased dependency on a single parents who were not able to access enough food for their families**.**

### Food adequacy

Food adequacy was affected in regard to quality, safety, and household food distribution. Inability to access food led to consumption of low-quality food which was inadequate to meet dietary needs. People’s goal was to fill the stomach irrespective of food quality and diversity. Reportedly, many households could only afford to eat one meal a day, mostly dinner [38]**.**

Moreover, they were uncertain about their next meal due to low purchasing power and unstable supply of food. As a result they rationed the foodstuff received from food-aid to save some for subsequent days. School closure also increased demands on household budgets and affected food distribution as some parents depended on school feeding programs to cut down their food expenditure. Additionally, others had to compromise by eating any type of available food despite having health conditions such as diabetes, hypertension which require modified dietary habits.

“...We have a food problem in Korogocho; if you have twenty shillings or thirty shillings you would use it to feed your family by buying superdip (powdered juice) and anyona, the bread (made of rejected breadcrumbs) that cost ten shillings (~$0.1)” (FGD, adult females, Korogocho).

**Fig. 3:**
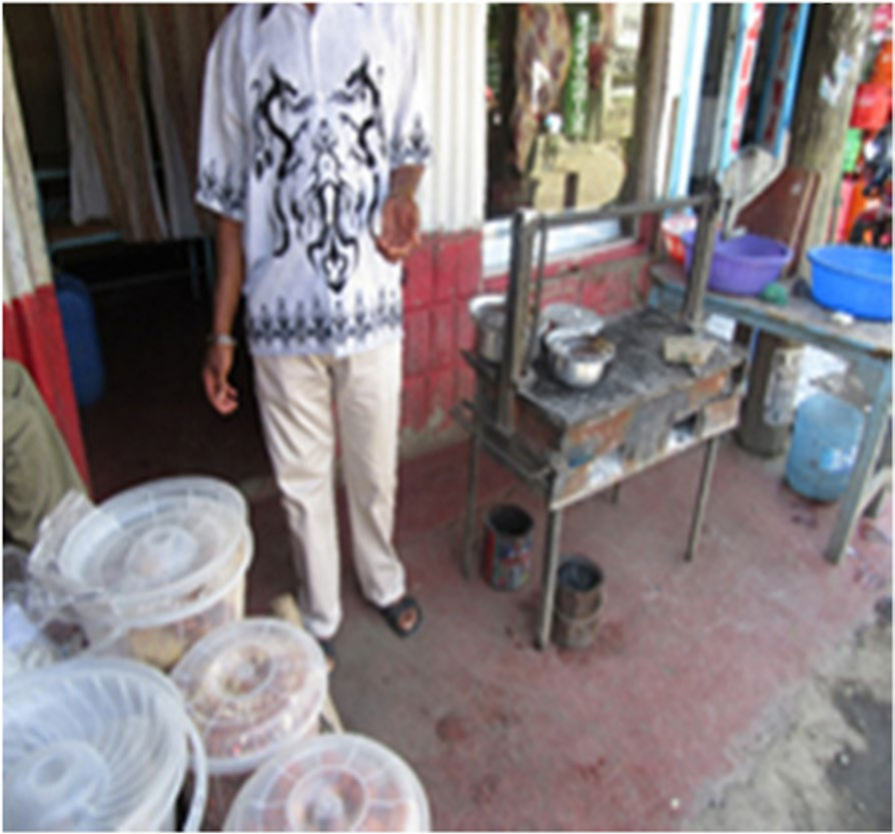
Photo 3 showing the situation of food adequacy in the markets in Nairobi slums during COVID19 lockdown period; Photo Credit: APHRC

Food quality and safety was also a problem as narrated by many of the participants. Low purchasing power reduced consumer demand for food from the traders who sell by the roadside or in small kiosks. Moreover, businesses were closed earlier than usual in compliance with curfew measures. Therefore, left-over cooked food was carried over to the next business day to reduce losses, with a corresponding reduction in the freshness of food consumed. Respondents recounted that some restaurants diluted the quality of food e.g., by increasing the amount of water “soup” in the food, so as to increase the amount of food available to meet customers’ demand Fig. 3**.**

“...The photo shows a challenge as there is food, but they are not eaten. So, it has affected the community because they don’t have money and that’s why the businesses people will sell their products for two days or a week and that is costly to them. And it is not even safe as we are supposed to eat fresh foods. But since there is no money people will not buy fresh foods and it’s not their liking...” (Photovoice, mixed group, Viwandani).

Some participants indicated that they were fed directly from the dumpsite by scavenging food or buying cheaper dumpsite food from the roadside vendors, which is mostly leftovers from the restaurants, disposed expired food from supermarkets, or rejects from industries. This resulted in consumption of low quality, expired, unhygienic and stale food.

“So, what we don’t always understand is that why are these foods always dumped here. So, if you eat them and you don’t get affected then you will make it a routine. So that’s what the people survived on” (KII religious leader Korogocho).

The situation of food insecurity in the community was reported to have led to an increase in the cases of malnutrition in the community, particularly in young children, as reported by the health care professionals.

“...It did affect their nutritional status. We had increased cases of malnutrition in the community especially amongst the under-fives...” (KII, PHN, Viwandani).

Over time the government through the media and local authorities has made efforts to provide means of promoting proper sanitation and hygienic practices and encouraging community members to boost immunity by eating a balanced diet and physical exercise. Likewise, participants recounted improved knowledge and awareness of healthy living and practices. However, during COVID-19 a balanced diet and physical activity was very unlikely due to food insecurity, lack of money and restrictions of movement, respectively.

“The government also helped us by asking us to do physical practice so that we could boost our immune systems and to try and balance our diets by eating a lot of fruits and greens” (FGD, adult females, Korogocho).

### Experiences regarding social protection measures and coping strategies for food

#### Social protection measures

The government and partners including non-governmental organizations (NGOs), the private sector, faith-based organizations and community-based organizations played a role in partially fulfilling the human right to food during the pandemic. This was through provision of food-aid, cash transfers to the most vulnerable households/individuals. Further, the government and partners provided free water supply, soap and sanitizers which helped curb the spread of the pandemic but also promoted food security in terms of promoting food hygiene. Some people also reported that they used the water to establish kitchen gardens.

“The food aid was quality because they would give out even a kilo of rice and a kilo of sugar and that would be good – also they would give out a kilo of beans so you would boil the rice and beans and that would be a good meal so even those who brought food aid really tried to give out balanced diet” (FGD adults male Korogocho).

“…We also had the government together with other partners putting in place hand washing points in various areas. We also had other partners coming in to provide cash transfers to most vulnerable patients like HIV and TB patients and those malnourished …” (KII, Public Health Nurse, Korogocho).

Similarly, from the digital storytelling, Mama Wangechi tells how she has benefited from social protection from the INGOs [38]:

“After a while, the village elder called me and helped me get funding from OXFAM Red cross. Within a month, I got the money, and I was very happy because I was among the lucky few, as some people did not get the money. I paid my rent and the food I had been taking on credit from the shop and the vendors. […] I am grateful especially to the Red Cross for giving me the money. Without them, I would not have opened my food vending business” (Mama Wangechi, digital storytelling speaker2).

In response to the pandemic the government introduced an economic empowerment program called “kazi mtaani” which targeted the jobless youth. The latter were enlisted into the program and assigned paid work within the community, mostly on environmental cleaning and sanitation. Community members appreciated this gesture.

“…Kazi mtaani has really assisted youths – not only those youths, but they also have parents, and some have really assisted their parents through the earning though kazi mtaani…” (KII, Senior Chief, Korogocho).

However, these social protection measures were short-lived, lasting only about 4 months, and their implementation was marred by challenges such as limited population coverage, irregularities, and discrimination in distribution. The use of police force by the government to ensure compliance of the measures met with a lot of criticism. Respondents reported brutal harassment and extortion in form of bribes where individuals were found or alleged not to have complied with the measures. In addition, some community leaders and chiefs were accused of injustices, corruption by requesting bribes to extend favors, unequal treatment, and unfairness in targeting and recruiting eligible members for food-aid and cash transfers. Consequently, vulnerable community members spent funds that they could have saved for food purchase in bribing the police and local authorities.

“There was some cash transfers that was supposed to be sent to the citizens, the truth of the matter is that the officials are the ones getting the cash transfers. When an administrative leader comes, they are bribed – when food is brought for distribution, the administrative leader takes most of it” (FGD youths, mixed group).

#### Coping strategies

**Fig. 4:**
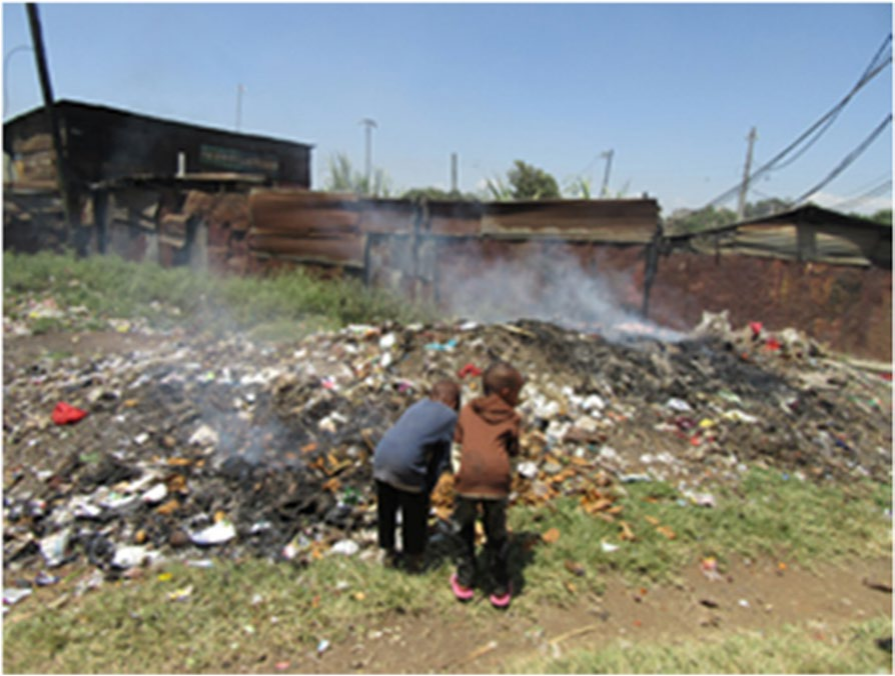
Photo 4 showing coping strategies with regards to food in Nairobi slums during COVID19 lockdown period; Photo Credit: APHRC

The disruption of sources of livelihood, compounded by the pre-existing socio-economic vulnerabilities of the members of informal settlement communities, forced them to adopt unpleasant coping strategies, including scavenging food from local dumpsites Fig. 4**.**

“...So, the children are at home, and they scavenge the dumpsite for food and if they find something else that they can sell they also go and sell it and get some money to buy something to eat. So all this is the effect of Corona virus which also caused lack of jobs...” (Photovoice, mixed group, Korogocho).

Skipping meals was also popular among the respondents. This resulted from reduction of the sources of income where the head of the household, and one or two other members had lost their livelihoods. Some resorted to having one meal a day, in a bid to reduce consumption and to save for the following day. They considered dinner (supper) as their most important meal, while others managed to eat two meals a day.

“So, it ended up that either we have breakfast and skip lunch so that we can have supper. So, food was a challenge and even now food is still a challenge in Korogocho” (IDI adult female Korogocho).

Participants suggested that limitations in sources of income resulted in socially undesirable or illegal alternative coping strategies such as child labor, sex work and theft. Vulnerability to food insecurity caused parents to engage their children in income generating activities such as hawking, which itself borders on child labor, a violation of fundamental human rights. Some were engaged in begging and scavenging activities. Young girls and married women were lured into sex work to earn income to purchase food. Some engaged in these activities with the consent from their parents. “So, you find that it’s very easy for the mother to give out their first-born daughter in order to feed the other young children”. It was reported that young people engaged in sex work for food. This led to teenage pregnancies, increase in sex work, child labor, substance use and school dropouts as narrated in the digital story telling by Mama Awiti (pseudonym), [39].

“.. Children decide to walk around and if they find an older man who promises them something, they will indulge into sex. So many girls got pregnant while boys started stealing. They started breaking into people’s homes and sell whatever they stole to get daily bread” (IDI youth female Korogocho).

Some community members depended on borrowing money from friends and Safaricom, a mobile phone company, which has a platform called “mshwari”, a saving and loaning service using the “MPESA” mobile money app, food donations sent by their relatives living in the rural areas whereas, others migrated back to their rural homes or sent their children to rural areas due to lack of enough money to sustain all household members. Additionally, some sough alternative sources of livelihood, including economic activities they had previously considered to be inferior such as doing laundry for others at a fee, hawking facial masks, prostitution, picking plastics and metals from dumpsites for sale and doing construction jobs for a lower pay due to low demand for labor.

Some community members adopted progressive coping strategies such as urban farming in order to produce food for their domestic consumption. They used a variety of innovative methods that for farming within their limited spaces. This strategy was also enhanced by the increased availability of water within informal settlements, a government strategy aimed at enhancing hygienic practices such as hand washing.

“...There were people who started thinking about farming… If you walk in Korogocho you will realize that there is somebody with something small like a garden – something small in most of the areas... I remember there was this group…–they even planted mushrooms…. So they decided to plant mushrooms and they were selling to people. There are also people who have kales within their residential places...” -KII, CHV, Korogocho “…. On my side, there was a time I was idle, and I didn’t have any work so I started doing some farming because we had water the whole time of the pandemic – so I grew some kales and spinach which would always support me. Whenever I had flour, I would just get kales or spinach from the farm and eat them…” (FGD, adult females, Korogocho).

Notably, some people embraced the spirit of ‘Ubuntu’ – I am because we are - and minded their neighbors through sharing whatever little they had.

“...whoever got food would share with the rest as they waited for the other distribution. So, the food was not distributed throughout…. so that’s why we made groups in our community, if people from this group received, they would share with the others and when they also received, they would also share” (IDI, adult females, Viwandani).

## Discussion

This study aimed to explore the lived experiences of vulnerable citizens in the informal settlements of Nairobi with regards to the human right to food, as guaranteed in the Kenyan Constitution 2010, following government’s COVID-19 response measures. Participants narrated that disruptions of the food supply chain and loss of livelihoods affected food availability, food access and affordability and adequacy. This consequently increased vulnerability to food and nutrition insecurity. The community employed various, sometimes unpleasant coping strategies. Despite the government putting in place some social protection measures, participants expressed negative perceptions as regards the government’s inability to respect, protect and adequately fulfil their right to food. A report on COVID-19 and food systems from the Indo-Pacific region indicated similar findings such as disrupted food supply chain owing to both international and local restrictions on logistics, significant loss of employment and incomes and food insecurity and resultant increases in food prices [40].

Various nations had taken similar measures to control the spread of COVID-19 [4]. However, many governments did not factor in adequate strategies to alleviate the effects of the restrictive measures particularly upon the most vulnerable populations [41, 42]. As a result, pandemic-related measures had similar impacts on food security in different countries across the globe [43]. Additionally, the measures exacerbated an ongoing economic crisis for the poor, reducing social interaction, and increasing discrimination and social stigma particularly as regards people returning home and health workers as reported in a similar study conducted in Nepal [44].

Though the response was intended to protect people from the corona virus, relevant measures were executed in ways that heightened vulnerabilities of the urban poor and worsened existing violations of human rights, including the right to adequate food and freedom from hunger [45]. For instance, studies in other countries suggested that social distancing contributed to socioeconomic hardships, loss of employment, and negative psychological effects. They were thus a difficult to sustain from an economic and financial perspective [46, 47]. Additionally, lockdown led to anxiety regarding food security, as conveyed in a study which used qualitative data polled from 12 countries [43]. Moreover, studies from sub-Saharan Africa and across the globe show that availability and accessibility of food has been limited by disruptionto the food chain resulting from lockdown and curfew measures taken by governments in many countries [48, 49].

Previous studies have also reported violation of human right to food before and after the pandemic in low, middleand high-income countries around the world [45, 50]. For instance, a study conducted in the United States reported violation of the human right to food in so far as mostly indigenous communities, people of color and those of lower socioeconomic backgrounds were struggling to put food on the table and were living paycheck to paycheck with limited support from the government [20]. Similarly a study in Uganda indicated that the state did not fulfil its obligation under the ICESCR to ensure children have their right to food and freedom from hunger [51].

It should be noted in addition that restrictions on the human right to freedom of movement owing to lockdown and curfew has limited enjoyment of other human rights, particularly the right to food [12]. Enjoyment of the right to food as stated in Article (Art) 43 [26] of the Kenyan constitution has been denied to many citizens during this pandemic [52]. Moreover, reports show that those charged with enforcing COVID-19 measures, such as national and county governments, as well as other agencies have violated human rights (e.g. liberty, access to food, and the ability to earn a livelihood), with the urban poor being at particular risk [29]. This notwithstanding the fundamental duty of the state to observe, respect, protect and fulfil the right to food, and in particular to ensure as a matter of priority that no one goes hungry (Art 21) [26].

The Committee on Socio-economic and Cultural Rights has authoritatively set out the principle of minimum core obligation which requires states to ensure basic provision of socio-economic rights (including food) and to work upwards towards the full realization of all the rights [53]. The Committee, by urging states to put in extra effort in protecting the socio-economic rights of marginalised and vulnerable groups such as those in informal settlements, even in the face of resource scarcity, seemed to have introduced safeguards against state neglect of this minimum core obligation, [54]. This set of tiered obligations applies in the case of emergencies, such as the COVID-19 pandemic, with particular reference to the needs of vulnerable groups such as those in informal settlements whose livelihoods were disrupted by the pandemic.

This study provides evidence to guide governments in responding appropriately and adequately to future emergencies especially with respect to more vulnerable groups. While in agreement with community leaders, the government’s COVID19 response measures may have been justifiable with respect to controlling the pandemic hence and thus the public interest in line with the provisions of the Kenyan Constitution [26]. However, such measures need to be necessary, proportionately tailored to their goal, and no more restrictive of fundamental rights than required by the emergency situation in line with the Constitution. Measures against COVID19 should be implemented in a way that protects, respects, and fulfils human rights, and upholds human dignity to the greatest extent possible. It is noteworthy that the government and partners instituted social protection measures to cushion vulnerable people from the impact of the response measures. However, the implementation of these social protection measures was marred with irregularities (unfair distribution of the resources by favoring specific individuals based on kin, friendship, and corruption/bribery) that further exacerbated vulnerability to some community members. Further, these social protection measures were notably short-lived as it was anticipated that the COVID19 pandemic would be a temporary problem. This calls for better emergency and disaster preparedness and response measures in Kenya.

There are a few limitations to this study. First is the use of a convenient sample, selected through purposive sampling technique that may introduce a selection bias. However, researchers included both youth and adults across the two study sites. Secondly, the socio-economic/demographic characteristics of 17 participants were not collected. These were mostly key informant respondents who were either community leaders, nurses, and church leaders. However the lack of information on the characteristics is unlikely to have led to bias, as the selection of the study participants was based on a group criterion e.g. status as community leader, health worker etc. and not on specific, individual characteristics e.g. age. Thirdly, the study was conducted in two of Nairobi’s urban informal settlements, therefore, the findings may not be generalized to the whole nation/larger urban centers because there were variations in the enforcement of the measures in different cities, differences in the environmental setup and household socio-economic levels. Hence, the perceived effects of restrictive measures taken against the spread of COVID-19 on human right to food may vary from one city to another and from one community to another. However, generalization to similarly impoverished low-income households’ vulnerable populations in urban slums is possible.

## Conclusion

The government of Kenya responded quickly in containing the spread of COVID-19. However, restrictive measures caused economic disruptions, disrupted the sources of already fragile livelihoods, and increased vulnerability. This exacerbated food insecurity and the vulnerability of the urban poor to hunger. In addition, measures taken by the government and other partners to cushion the most affected people, including cash transfers and food distribution were inadequate, marred by corruption and inequality, unsustainable and only benefited a section of the population. Therefore, in future, targeting and recruitment systems should be better coordinated and further improved to reach the majority of those who are needy/vulnerable. Moreover, some of the response measures were implemented in a way that caused violations of the human right to food. Consequently, response measures to pandemics and other misfortunes should be human-centered and executed in ways that guarantee protection of human rights. The pandemic has exposed the fragility of the urban food system. Hence, there is need to strengthen the urban food system to make it more resilient to external shocks.

## Data Availability

The datasets used and/or analysed during the current study are available from the corresponding author on reasonable request.
